# Ligation of Signal Inhibitory Receptor on Leukocytes-1 Suppresses the Release of Neutrophil Extracellular Traps in Systemic Lupus Erythematosus

**DOI:** 10.1371/journal.pone.0078459

**Published:** 2013-10-18

**Authors:** Kristof Van Avondt, Ruth Fritsch-Stork, Ronald H. W. M. Derksen, Linde Meyaard

**Affiliations:** 1 Department of Immunology, Laboratory of Translational Immunology, University Medical Center Utrecht, Utrecht, The Netherlands; 2 Department of Rheumatology and Clinical Immunology, Laboratory of Translational Immunology, University Medical Center Utrecht, Utrecht, The Netherlands; University of São Paulo, Brazil

## Abstract

Neutrophil extracellular traps (NETs) have been implicated in the pathogenesis of systemic Lupus erythematosus (SLE), since netting neutrophils release potentially immunogenic autoantigens including histones, LL37, human neutrophil peptide (HNP), and self-DNA. In turn, these NETs activate plasmacytoid dendritic cells resulting in aggravation of inflammation and disease. How suppression of NET formation can be targeted for treatment has not been reported yet. Signal Inhibitory Receptor on Leukocytes-1 (SIRL-1) is a surface molecule exclusively expressed on phagocytes. We recently identified SIRL-1 as a negative regulator of human neutrophil function. Here, we determine whether ligation of SIRL-1 prevents the pathogenic release of NETs in SLE. Peripheral blood neutrophils from SLE patients with mild to moderate disease activity and healthy donors were freshly isolated. NET release was assessed spontaneously or after exposure to anti-neutrophil antibodies or plasma obtained from SLE patients. The formation of NETs was determined by microscopic evaluation using DNA dyes and immunostaining of NET components, as well as by live cell imaging. We show that SLE neutrophils spontaneously release NETs. NET formation is enhanced by stimulation with antibodies against LL37. Inhibition of nicotinamide adenine dinucleotide phosphate (NADPH) oxidase activity and MEK-ERK signaling prevents NET release in response to these antibodies. Signaling via the inhibitory receptor SIRL-1 was induced by ligation with anti-SIRL-1 specific antibodies. Both spontaneous and anti-neutrophil antibody-induced NET formation is suppressed by engagement of SIRL-1. Furthermore, NET release by healthy neutrophils exposed to SLE plasma is inhibited by SIRL-1 ligation. Thus, SIRL-1 engagement can dampen spontaneous and anti-neutrophil antibody-induced NET formation in SLE, likely by suppressing NAPDH oxidase and MEK-ERK activity. Together, these findings reveal a regulatory role for SIRL-1 in NET formation, potentially providing a novel therapeutic target to break the pathogenic loop in SLE.

## Introduction

Systemic lupus erythematosus (SLE) is a chronic relapsing-remitting autoimmune disease with pleiotropic, at times life-threatening, clinical manifestations. SLE has a prevalence of 20 to 150 people per 100,000 individuals. The disease is characterized by a permanent state of immune stimulation, leading to the accumulation of autoantibodies targeting double-stranded DNA (dsDNA) as well as other nuclear antigens. The presence of type I interferon-producing plasmacytoid dendritic cells is a hallmark of SLE [1]. Moreover, neutrophils have recently received attention as these cells can form neutrophil extracellular traps (NETs) which may serve as a source of autoantigens and be involved in diverse disease manifestations, especially nephritis [2-5].

SLE patients produce autoantibodies against antimicrobial peptides present in NETs such as human neutrophil peptide (HNP) and the antimicrobial peptide LL37 [2]. Exposure to these autoantibodies in turn stimulates neutrophils from SLE patients to release NETs which gives the immune system access to antigenic DNA resulting in perpetuation or even aggravation of disease. Though the molecular events that control the formation of NETs are largely unknown, a role for the nicotinamide adenine dinucleotide phosphate (NADPH) oxidase was suggested in the induction of NETosis by anti-ribonucleoprotein (RNP) antibodies of SLE patients [3]. How suppression of NET release can be exploited as a treatment strategy remains to be determined [6].

The inhibitory receptor Signal Inhibitory Receptor on Leukocytes-1 (SIRL-1) is an immunoreceptor tyrosine-based inhibitory motif (ITIM)-bearing membrane protein expressed by human phagocytes [7]. SIRL-1 is capable of recruiting Src homology 2 domain-containing tyrosine phosphatases SHP-1 and SHP-2 and functions as a negative modulator of innate immune cell effector mechanisms. Engagement of SIRL-1 dampens signaling of the MEK-ERK pathway, resulting in suppressed FcR-mediated generation of reactive oxygen species (ROS) [8].

Given the role of SIRL-1 as a suppressor of neutrophil function and the new perspective that dysregulated NET formation perpetuates SLE pathogenesis, we reasoned that SIRL-1 could control the release of NETs in SLE. Here, we show that SIRL-1 ligation suppresses NET formation by peripheral neutrophils from SLE patients and healthy neutrophils stimulated with anti-neutrophil antibodies. We also demonstrate that engagement of SIRL-1 can inhibit the release of NETs by healthy neutrophils exposed to SLE plasma.

## Materials and Methods

### Patient information

This study was undertaken after the approval of the Medical University of Utrecht institutional review board. All patients and healthy controls gave written informed consent. Seventeen patients meeting the ACR criteria for SLE [9] were enrolled in the study. Sixteen patients were female (94%). Sex-matched healthy controls were used. Disease activity was measured according to the SELENA-SLEDAI score at the day of blood collection [10]. Patients had mild to moderate disease activity with the SELENA-SLEDAI ranging from 0-8 and a mean SLEDAI of 3.6 (± 2 SD). Mostly, disease activity consisted of an elevated titer of dsDNA antibodies. Specific patient characteristics including medication are listed in Table 1.

**Table 1:** Patient characteristics and ongoing treatment*.

**Age**	**Gender**	**SLEDAI**	**Disease activity at visit: organ**	**dsDNA (IU/ml)**	**ENA**	**Medication**	**History of nephritis**
46	F	8,0	serology, alopecia, kidney (dysmorphic erythrocytes)	75,0	SS-A, SS-B, nucleosomes, PM-Scl	HCQ, Aza, Pred (10mg)	yes
46	F	2,0	serology	0,0	SS-A, SS-B	HCQ	no
33	F	2,0	serology	100,0	none	none	no
53	F	2,0	serology	80,0	none	HCQ, Aza, Pred (10mg)	yes
39	F	2,0	serology	12,0	none	HCQ, Pred (7,5mg)	no
39	F	7,0	serology, hematology, skin	200,0	SS-A, histones, nucleosomes, Sm, RibP	HCQ, Aza, Pred (5mg)	no
58	F	0,0	none	0,6	SS-A	HCQ, Aza, Pred (5mg)	yes
32	F	4,0	serology, skin	25,0	SS-A, SS-B, histones, Sm, RibP	Aza, Pred (5mg)	yes
51	F	6,0	serology, kidney (dysmorphic erythrocytes)	1,8	none	Aza, Pred (5mg)	yes
36	F	4,0	serology	65,0	none	Aza, Pred (10mg)	yes
53	F	3,0	serology, hematolgoy	8,9	SS-A, SS-B	HCQ	no
27	F	5,0	serology, hematology, skin	3,6	SS-A	HCQ	no
55	F	4,0	serology	22,0	none	MMF, Pred (10mg)	yes
30	F	2,0	serology	39,0	none	Pred (5mg)	yes
21	F	4,0	serology	75,0	histones, Sm	HCQ, Aza, Pred (10mg)	yes
52	M	3,0	serology, hematology	8,9	none	HCQ, Aza, Pred (10mg)	no
51	F	4,0	serology	16,0	SS-A, nucleosomes	HCQ, MTX (15mg)	no

* SLEDAI: SLE disease activity index, dsDNA: double-stranded DNA, ENA: extractable nuclear antigens, Sm: Smith antigen, SS-A: Sjoegren Syndrome Antigen A, SS-B: Sjoegren Syndrome Antigen B, PM-Scl: Polymyositis-Scleroderma, RibP: Ribosomal antigen P, HCQ: Hydroxychloroquine, Aza: Azathioprine, Pred: Prednisone, MMF: Mycophenolate Mofetil, MTX: Methotrexate

### Neutrophil isolation

Human normal-density neutrophils were isolated from heparinized venous blood of healthy donors or SLE patients by density gradient centrifugation with Histopaque 1119 (Sigma Aldrich) and Ficoll (Amersham Biosciences). Plasma was collected and used for stimulation of neutrophils. If not stated otherwise, cells were resuspended in RPMI 1640 medium supplemented with 2% heat-inactivated FCS. Neutrophils (5 x 10^5^) were seeded on uncoated glass for live cell imaging or glass coverslips for fluorescence microscopy pretreated with 0.001% poly-L-lysine (Sigma Aldrich). Incubations were performed at 37°C in the presence of 5% CO_2_.

### Stimulation and inhibition of NET formation

Neutrophils were stimulated with anti-LL37 (10 µg/ml; Hycult biotech), anti-HNP (10 µg/ml; Novus Biological), 10 µg/ml irrelevant surface-binding control antibodies (anti-MHCI; in-house) or 25 ng/ml PMA (Sigma Aldrich) as described previously [2]. Previous experiments (results not shown) confirmed that the use of irrelevant surface-binding control antibodies did not differ from that of nonbinding isotype-matched IgG1 (eBioscience). Plasma from SLE patients and healthy controls was used at a concentration of 20% to induce NET formation. In some experiments, neutrophils were incubated with inhibitors 30 min before stimulation. The NADPH oxidase inhibitor diphenylenoiodonium (DPI; Sigma Aldrich) was used at 10 µM and the MEK inhibitor U0126 (Cell Signaling Technology) at 50 µM. DMSO was used as vehicle control. Where indicated, neutrophils were incubated at 4°C with 10 µg/ml anti-SIRL-1 (clone 1A5) or irrelevant control antibodies followed by 20 µg/ml goat anti-mouse F(ab’)_2_ fragments (SouthernBiotech) for 30 min prior to the induction of NET formation.

### Quantification of extracellular NET-DNA

Cells were stimulated to induce the release of NETs. After 3 h, cells were stained with Sytox Green (0.5 µM; Invitrogen), a cell-impermeable DNA dye, gently washed, fixed with 4% paraformaldehyde (PFA) and stained with Hoechst 33342 (1 µM; Invitrogen). For quantification of NET release, at least 4 fields of view (each 659 x 659 µm) per condition were captured using a 20x objective lens. NETs were quantified using previously described methodology [11]. In short, the area of positive Sytox staining for each microscopic field was measured by analyzing the images using ImageJ software (NIH). Contrast was adjusted to minimize background autofluorescence and a fluorescent threshold was set to result in positive staining only. The same contrast and fluorescence threshold were applied to all images from all conditions within the experiment. The Sytox-positive pixel counts were divided by the total number of pixels of thresholded 8-bit images using ImageJ software, and expressed as the percentage of image area covered by positive fluorescence staining in each field of view.

### Immunostaining and quantification of released NET constituents

Neutrophils were seeded on glass coverslips treated with 0.001% poly-L-lysine, allowed to settle, and treated with anti-LL37 antibodies (10 µg/ml) or irrelevant control antibodies (10 µg/ml). Myeloperoxidase (MPO) and neutrophil elastase (NE) were immunostained as described elsewhere [12]. After 3 h, cells were fixed with 4% PFA, permeabilized with 0.25% Triton X-100 in PBS, blocked (1% BSA and 0.1% Tween 20 in PBS) and incubated overnight with primary antibodies anti-MPO (ab45977; Abcam); anti-NE (sc-9518; Santa Cruz Biotechnology), which were detected with F(ab’)_2_ fragments of secondary antibodies coupled to Alexa Fluor 488 (Molecular Probes) or DyLight 594 (Jackson ImmunoResearch Laboratories). For DNA detection, Hoechst 33342 was used. Specimens were mounted in Fluoromount-G (SouthernBiotech) and analyzed with a UPlanSApo 20x/0.75 air objective on a widefield inverted microscope (IX71; Olympus). To quantify the release of NET constituents, the same fluorescence threshold was applied to all images from all conditions within the experiment. The MPO-stained (green channel) and NE-stained (red channel) areas were measured using ImageJ software.

### Microscopy

Fixed cells were imaged using an Olympus IX71 widefield inverted microscope with a UPlanSApo 20x/0.75 air objective in Fluoromount-G (SouthernBiotech). Two or three of the appropriate band pass excitation filters (360/40, blue channel; 490/20, green channel or 555/28, red channel) were used in succession and fluorescence in blue, green and red channels was visualized with the appropriate emission filters. To minimize differences in fluorescence, the same exposure times for excitation filters were applied between experiments. Typical exposure times for fluorescence channels are as follows. For immunostaining of NET components: blue channel (DNA, 150 ms), green channel (MPO, 250 ms) and red channel (NE, 200 ms). For quantification of extracellular NET-DNA: blue channel (Hoechst, 200 ms), green channel (Sytox Green, 100 ms). Fluorescence was detected using a Photometrics EMCCD 1024 x 1024 pixel camera and Softworx acquisition software. Images were processed using ImageJ.

### Flow cytometry

For measurement of SIRL-1 expression, human neutrophils (1 x 10^5^) in PBS supplemented with 10% BSA were stained with anti-SIRL-1 or isotype-matched control antibodies conjugated to FITC, in the dark for 30 min at 4°C. Neutrophils were then washed twice and finally resuspended in PBS supplemented with 10% BSA. Fluorescence was read on a BD FACS Calibur flow cytometer (BD Biosciences) and analyzed using CellQuest software (BD Immunocytometry Systems).

### Live cell imaging

NET release was followed as described elsewhere [13]. In short, 2-5 x 10^5^ neutrophils were labelled with 1 µM Hoechst 33342 in RMPI 1640 medium (phenol red-free) supplemented with 10 mM Hepes and seeded into culture plates equipped with glass bottoms (Mattek). To record the presence of extracellular DNA, the medium contained Sytox Green at a concentration of 0.5 µM. Neutrophils were stimulated with 20% SLE plasma and monitored at 37°C on a microscope (LSM720; Carl Zeiss) with a Plan-Neofluar 40x/0.6 objective over a period of 3 h. Every minute, a set of two images (blue and green fluorescence) was obtained. Laser excitation at 405 and 488nm was used in succession and fluorescence in blue and green channel was visualized with the appropriate filters. Exposure time for both wavelengths was 60 ms. The system was controlled by the Zen 2009 software (Carl Zeiss). Individual frame overlays and videos were processed using ImageJ software.

### Statistical analysis

Measurements were analyzed in GraphPad Prism 5. Paired Student’s *t* test or Student’s *t* test were used to compare 2 samples. The comparison of 3 samples was performed by ANOVA and Dunnett’s multiple comparison tests. Correlation was established by two-tailed Pearson’s correlation test. Correlation coefficient (r^2^) and significance (p) are given in the figure.

## Results

### Anti-LL37 antibody-induced NET formation depends on NADPH oxidase activity and MAPK signaling

**Figure 1:**
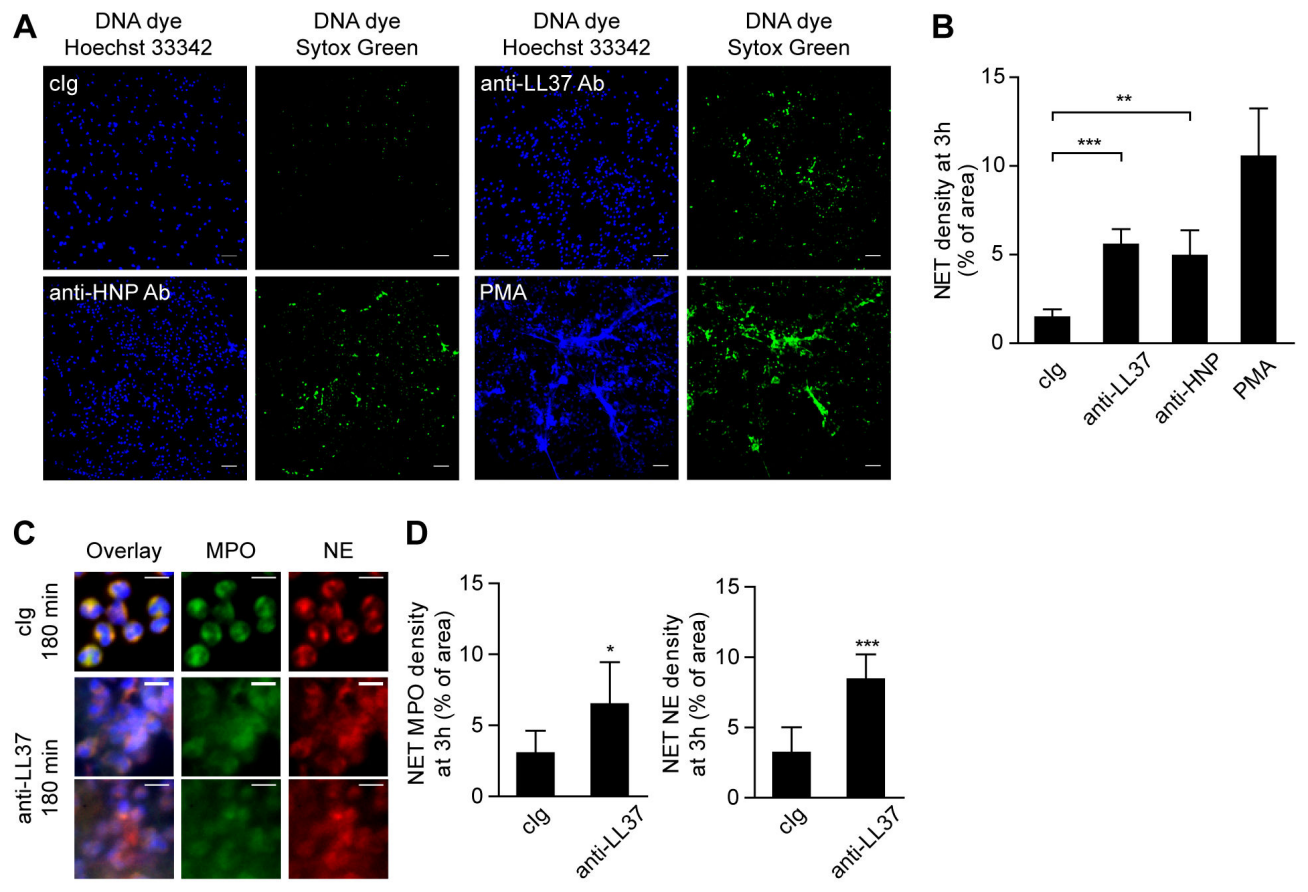
Localization of MPO and NE on extracellular DNA upon NET release in response to anti-neutrophil antibodies. A) Fluorescence imaging of healthy neutrophils cultured with 10 µg/ml control IgG, 10 µg/ml anti-LL37 antibody, 10 µg/ml anti-HNP antibody or 25 ng/ml PMA as positive control and stained for total DNA with Hoechst 33342 (left panels) and extracellular DNA (Sytox Green, right panels) after 3 h of incubation. Scale bars, 50 µm. B) Quantification of NET release by healthy neutrophils using fluorescence microscopy. The density of extracellular DNA (stained with Sytox Green) over the image area after 3 h of incubation with control IgG (n=4), anti-LL37 antibody (n=4), anti-HNP antibody (n=3) or PMA (n=3) is shown as mean±SD. **p<0.01; ***p<0.001, ANOVA (adjusted for Dunnett’s test). C) Immunostaining for NET components (green, myeloperoxidase (MPO); red, neutrophil elastase (NE); blue, DNA). The experiment was repeated three times with neutrophils from independent donors, with similar results. Scale bars, 10 µm. Figures show details of larger fields of view, that were used to quantify NET NE and NET MPO density. Original magnification 20x. D) Quantification of NET release using fluorescently conjugated NET-specific antibodies (either anti-MPO or anti-NE antibodies). The MPO- and NE-stained areas are shown as mean±SD (n=3). *p=0.0498; ***p=0.0006, Student’s *t* test.

Upon incubation of peripheral blood neutrophils from healthy donors with anti-neutrophil antibodies against LL37 and HNP, we observed significant formation of NETs, while no extracellular DNA was released when neutrophils were incubated with control IgG (Figure 1A and B).

Myeloperoxidase (MPO) and neutrophil elastase (NE) are granular proteins that associate with NETs [12,14,15]. The presence of decondensed extracellular DNA that stains positively for these granular markers is consistent with the process of NET formation and is distinct from necrosis. Immunostaining revealed that none of these NET constituents were released when cells were incubated for 3 h with irrelevant control IgG, while the nuclei retained a normal, condensed shape (Figure 1C, top panels). After stimulation with anti-LL37 antibodies for 3 h (Figure 1C, middle and bottom panels), staining for nuclear and granular components was no longer clearly separated and neutrophils released extracellular structures where NE, MPO and DNA colocalize. Quantification of MPO- and NE-stained areas confirmed that significant release of MPO and NE occurred in response to anti-LL37 antibodies (Figure 1D), similar to the release of extracellular DNA stained with Sytox Green (Figure 1B).

**Figure 2:**
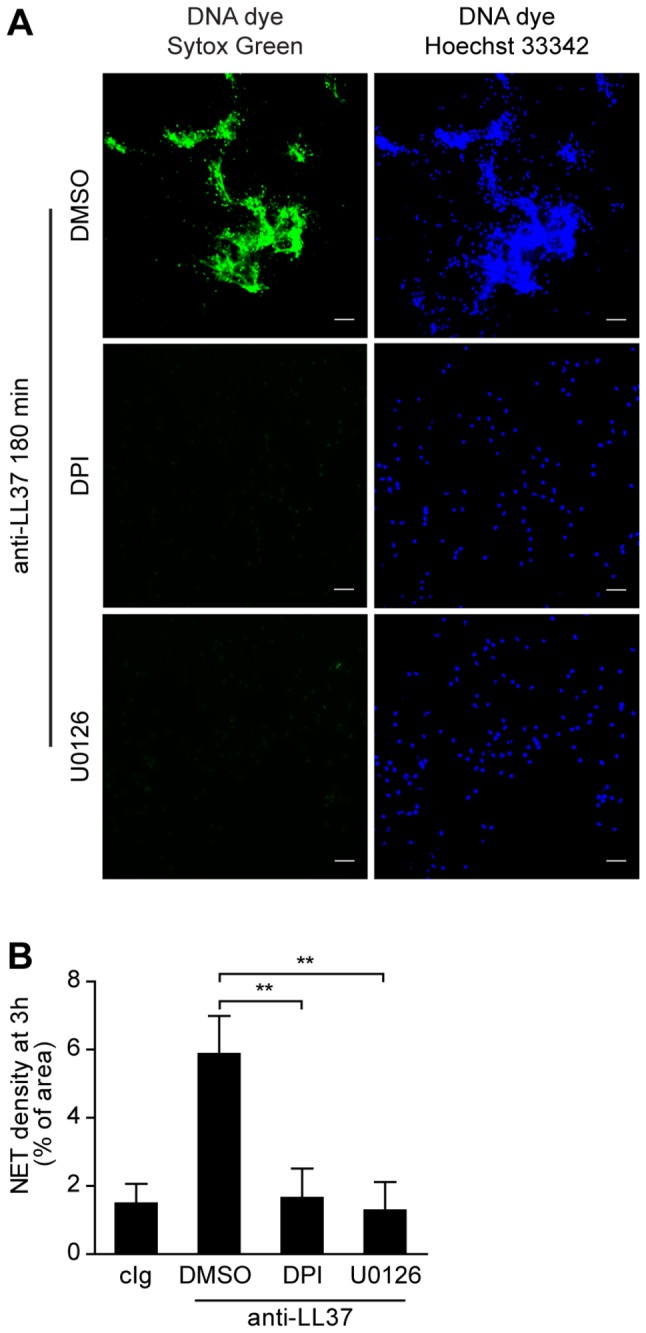
NADPH oxidase activity and MAPK signaling are required for NET formation in response to antibodies against LL37. Healthy neutrophils were pretreated for 30 min with or without the NADPH oxidase inhibitor DPI (10 µM) or U0126 (50 µM), a specific mitogen-activated protein/extracellular signal-regulated kinase kinase (MEK) inhibitor, before incubation with anti-LL37 antibodies for 3 h. DMSO was used as vehicle control. A) One out of three independent experiments is shown. NET release is determined by staining for DNA and visualized by fluorescence microscopy. Scale bars, 50 µm. B) The amount of NET-DNA is quantified as in Figure 1B. Mean±SD of three independent donors is indicated. **p<0.01, ANOVA (adjusted for Dunnett’s test).

NET release induced by anti-RNP antibodies derived from SLE patients was previously shown to depend on the formation of ROS [3]. We now show that pharmacological inhibition of NADPH oxidase activity by incubation with DPI also blocks NET release by neutrophils stimulated with antibodies against LL37 (Figure 2A and B). MAPK signaling is often involved in activation of the NADPH oxidase. Moreover, the MEK-ERK signaling pathway has been implicated in NET release triggered by PMA [16]. Neutrophils did not release NETs in the presence of U0126 (Figure 2A and B), a selective inhibitor of MEK activity, demonstrating the involvement of specific cellular signaling in this process of neutrophil death. Thus, activity of the NADPH oxidase and MEK-ERK sinaling are required for anti-LL37 antibody-induced NET formation.

### Engagement of SIRL-1 suppresses anti-LL37 antibody-induced NETosis in healthy controls and individuals with SLE

**Figure 3:**
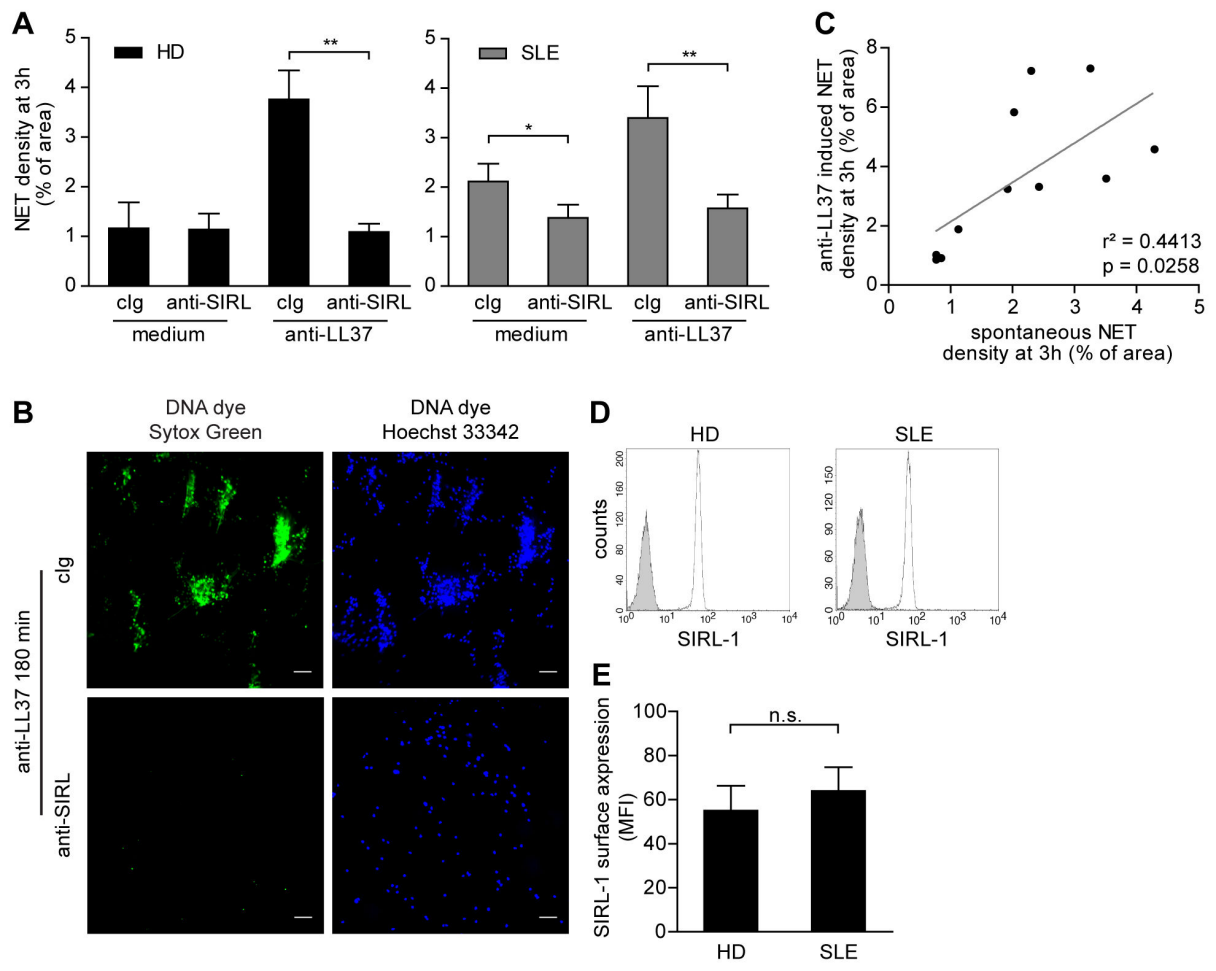
Ligation of SIRL-1 suppresses both spontaneous and anti-LL37-triggered NET release by SLE neutrophils. A) Healthy (HD) or SLE neutrophils were incubated with anti-LL37 antibodies with or without pretreatment with anti-SIRL-1 mAb. NET-DNA release was quantified after 3 h in multiple experiments by fluorescence microscopy to reflect the area covered by extracellular DNA (as described in Figure 1B). HD, mean±SEM of four independent donors is shown. **p=0.0014, paired Student’s *t* test. SLE, mean±SEM is shown (medium, n=11; anti-LL37, n=13). *p=0.0117; **p=0.0052, paired Student’s *t* test. B) Representative images of anti-LL37-stimulated SLE neutrophils with or without SIRL-1 ligation stained for extracellular DNA (Sytox Green, left panels) and total DNA (Hoechst 33342, right panels). Scale bars, 50 µm. C) Correlation of spontaneous and anti-LL37 antibody-induced NET formation by neutrophils from individuals with SLE. r^2^=0.4413; p=0.0258, Pearson’s correlation test. D) Flow cytometry analysis of SIRL-1 surface expression on freshly purified neutrophils from healthy donors and SLE patients. Representative histograms (grey filled, isotype-matched antibody; white dotted, anti-SIRL-1 mAb) are shown. E) Results from multiple independent donors (HD; n=6, SLE; n=7) are given. Average mean fluorescence intensity of each group±SD is indicated, Student’s *t* test.

As we previously showed that engagement of SIRL-1 abrogates signaling of the MEK-ERK pathway and suppresses the formation of ROS in neutrophils [8], we hypothesized that SIRL-1 dampens the release of NET-DNA in response to antibodies targeting LL37. Indeed, ligation of SIRL-1 with mAbs on the surface of neutrophils from healthy controls before addition of antibodies against LL37 suppressed NET formation to background levels (Figure 3A).

Neutrophils isolated from SLE patients with active disease were shown to be more prone to undergo NETosis than healthy neutrophils [2]. In our group of SLE patients (Table 1) with mild to moderate disease activity, 4 out of 13 individuals showed increased NET release in response to anti-LL37 antibodies compared to controls (Figure 3A). The extent of NET induction differed between patients. SIRL-1 ligation dampened NET formation by SLE neutrophils in response to anti-LL37 antibodies by 54% (p=0.0052) (Figure 3A and B). Furthermore, SLE neutrophils from this mild to moderate patient group spontaneously released increased amounts of NETs without *in vitro* stimulation (Figure 3A). In our study, we were able to establish a positive correlation (r^2^=0.4413; p=0.0258) between the level of NETosis at baseline and the amount of NET-DNA released in response to antibodies targeting LL37 (Figure 3C). Importantly, engagement of SIRL-1 on the surface of SLE neutrophils also reduced the amount of spontaneous NET formation by *ex vivo* neutrophils from SLE patients by 35% (p=0.0117) (Figure 3A).

We found no difference in the surface expression of SIRL-1 between healthy and *ex vivo* SLE neutrophils (Figure 3D and E), supporting the idea of targeting SIRL-1 as a potential way of suppressing neutrophils and the release of NETs in SLE.

### Ligation of SIRL-1 prevents the release of NET-DNA in response to plasma from SLE patients

**Figure 4:**
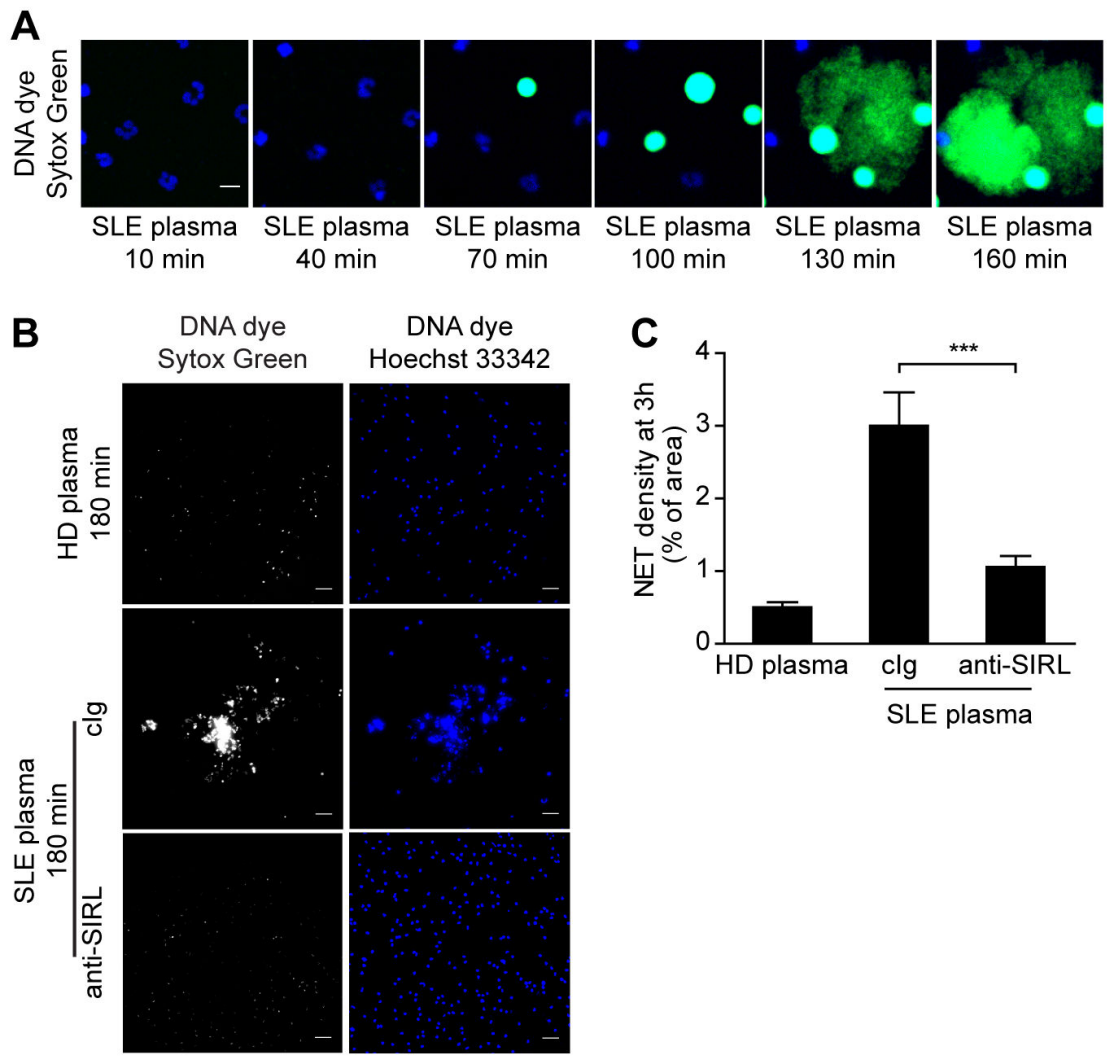
Engagement of SIRL-1 prevents NETosis in response to SLE plasma. A) NET release from healthy neutrophils exposed to SLE plasma visualized as extracellular DNA (Sytox Green) by live cell imaging (Video S1) (neutrophils are blue; NETs are green). Individual frames are shown. Scale bar, 10 µm. B) Representative images of netting healthy neutrophils in response to SLE plasma with or without ligation of SIRL-1. Scale bars, 50 µm. C) Results from multiple independent donors are given. Neutrophils from 4 healthy donors were cultured for 3 h with 20% plasma from either nonautologous healthy controls (n=4) or individuals with SLE (n=5 or 6) in the presence or absence of anti-SIRL-1 mAb. NET formation was analyzed by fluorescence microscopy and the amount of NET-DNA quantified. Average density of each pooled group±SEM is shown. ***p<0.0001, paired Student’s *t* test.

Given the previously reported presence of NET-inducing autoantibodies circulating in SLE patients [2], we sought to determine whether engagement of SIRL-1 on healthy neutrophils suppresses the release of NETs in response to plasma from SLE patients. Exposure of healthy neutrophils to SLE plasma in the presence of DNA dyes revealed the formation of NETs (Figure 4A and Video S1). Importantly, SIRL-1 ligation on the surface of healthy neutrophils prevented the induction of NETosis in response to SLE plasma by 59 to 76%, depending on the neutrophil donor (Figure 4B and C).

## Discussion

Despite the recognition of the contribution of NETs to autoimmunity, suppression of NET formation in pathologic settings remains largely unaddressed. Previously, NET formation was shown in paediatric SLE and in adults with active disease [2,3]. Here, we show that also in SLE patients with mild to moderate disease activity, neutrophils release NETs spontaneously and upon incubation with anti-neutrophil antibodies. Most importantly, we describe for the first time a role for SIRL-1, an ITIM-bearing inhibitory receptor, in the regulation of immunogenic NET release by primary human neutrophils in SLE.

In our study, we used antibodies targeting LL37 as a NET-inducing stimulus in most experiments, since autoantibodies against LL37 were shown to circulate in SLE patients and to stimulate isolated neutrophils to release NETs [2]. We now show that engagement of the inhibitory receptor SIRL-1 strongly prevents the formation of NETs by SLE neutrophils upon exposure to these antibodies. Importantly, NET release induced by SLE plasma was also suppressed by SIRL-1 ligation, albeit slightly less efficient, possibly due to other factors in plasma. Plasma-induced NET formation may mimic the positive feed-back loop occurring in patients with SLE, and underscores the potential of the regulation of pathogenic NET release through engagement of SIRL-1. The endogenous molecule that binds SIRL-1 is not known to date. Insights into the identity of this ligand might further broaden our understanding on the role of SIRL-1 in ensuring a normal physiological balance.

In SLE, neutrophils do not circulate as a single population. Recent work highlights the presence of a unique subset of neutrophils in SLE patients, named low-density granulocytes (LDGs). In particular, Villanueva et al. reported that LDGs show increased ability to release NETs [4]. Whether ligation of SIRL-1 suppresses the formation of NETs by other neutrophil subsets remains to be determined and should be the focus of future research.

Our current experiments demonstrate that NET formation in response to anti-LL37 antibodies is dependent on NADPH oxidase. Binding of antibodies to LL37 may cause the formation of immune complexes on the surface of neutrophils. Garcia-Romo et al. showed that NETosis induced by anti-RNP antibodies depends on binding to FcγRIIa and the formation of ROS [3]. Our findings support the common involvement of this enzyme complex in NET formation in response to anti-neutrophil antibodies. Interestingly, in earlier studies, we demonstrated SIRL-1 to suppress FcR-mediated ROS generation [8]. SIRL-1 may thus suppress NET formation by negatively regulating FcR-induced NADPH oxidase activity. In the present study, we also observed the requirement for MEK-ERK signaling in anti-LL37 antibody-induced NET release, which was previously shown to be involved in PMA-induced NET formation [16]. Our data further establish an important role for this pathway in NET signaling. Previously, we showed that ligation of SIRL-1 results in suppression of this signaling pathway triggered by FcR crosslinking. Keshari et al. recently suggested that ERK activation during PMA-induced NET release occurred downstream of the generation of ROS [17]. Whether SIRL-1 signaling directly regulates ERK activation or via inhibition of NADPH oxidase activity remains to be determined.

Crucial roles for MEK-ERK signaling, NE and ROS in the molecular events leading to the release of NETs have been proposed. Although likely not specific, molecules interfering with these subcellular mechanisms might represent potential strategies to modulate NET formation. Alternatively, inhibition of NET formation by specifically targeting molecules on the neutrophil surface may be considered in the treatment of SLE. NETs have been implicated in the pathology of SLE nephritis [5], but may play a role in several other SLE manifestations as well. As NETs induce endothelial damage [4], a role for NETs in premature atherosclerosis seen in SLE patients is probable. Thus, our findings may form the basis of novel therapeutic intervention strategies through SIRL-1 in SLE. To evaluate the true potential of SIRL-1 ligation to modulate NET release and abrogate autoimmune responses and tissue damage *in vivo*, the generation of a mouse model would proof to be a valuable tool. Also, the development of molecules that specifically interfere with SIRL-1 signaling will become indispensable.

## Supporting Information

Video S1 **NETs are released by healthy neutrophils in response to SLE plasma.** Time lapse of NET formation (related to Fig 4A in which individual frames are shown) shows that NETs detected with Sytox Green, a cell-impermeable, DNA-specific dye, are released when neutrophils are exposed to SLE plasma. Using fluorescence microscopy neutrophils (blue – labelled with Hoechst 33342) were visualized releasing NET-DNA (green - dyed with Sytox Green) within 3 h.
(MOV)
